# Comparative Theoretical Studies on a Series of Novel Energetic Salts Composed of 4,8-Dihydrodifurazano[3,4-b,e]pyrazine-based Anions and Ammonium-based Cations

**DOI:** 10.3390/molecules24183213

**Published:** 2019-09-04

**Authors:** Binghui Duan, Ning Liu, Bozhou Wang, Xianming Lu, Hongchang Mo

**Affiliations:** 1 Xi’an Modern Chemistry Research Institute, Xi’an 710065, China; 2 State Key Laboratory of Fluorine & Nitrogen Chemicals, Xi’an 710065, China

**Keywords:** difurazano[3,4-*b*,*e*]pyrazine, ionic salts, detonation performance, impact sensitivity, thermodynamics of formation

## Abstract

4,8-Dihydrodifurazano[3,4-*b*,*e*]pyrazine (DFP) is one kind of parent compound for the synthesis of various promising difurazanopyrazine derivatives. In this paper, eleven series of energetic salts composed of 4,8-dihydrodifurazano[3,4-*b*,*e*]pyrazine-based anions and ammonium-based cations were designed. Their densities, heats of formation, energetic properties, impact sensitivity, and thermodynamics of formation were studied and compared based on density functional theory and volume-based thermodynamics method. Results show that ammonium and hydroxylammonium salts exhibit higher densities and more excellent detonation performance than guanidinium and triaminoguanidinium salts. Therein, the substitution with electron-withdrawing groups (–NO_2_, –CH_2_NF_2_, –CH_2_ONO_2_, –C(NO_2_)_3_, –CH_2_N_3_) contributes to enhancing the densities, heats of formation, and detonation properties of the title salts, and the substitution of –C(NO_2_)_3_ features the best performance. Incorporating N–O oxidation bond to difurazano[3,4-*b*,*e*]pyrazine anion gives a rise to the detonation performance of the title salts, while increasing their impact sensitivity meanwhile. Importantly, triaminoguanidinium 4,8-dihydrodifurazano[3,4-*b*,*e*]pyrazine (J4) has been successfully synthesized. The experimentally determined density and *H*_50_ value of J4 are 1.602 g/cm^3^ and higher than 112 cm, which are consistent with theoretical values, supporting the reliability of calculation methods. J4 proves to be a thermally stable and energetic explosive with decomposition peak temperature of 216.7 °C, detonation velocity 7732 m/s, and detonation pressure 25.42 GPa, respectively. These results confirm that the derivative work in furazanopyrazine compounds is an effective strategy to design and screen out potential candidates for high-performance energetic salts.

## 1. Introduction

Very recently, there has been a growing interest in the development of nitrogen-rich heterocycle energetic compounds (NHEC). These high nitrogen energetic materials feature attractive characteristics including high density, insensitivity to external stimuli, and environmentally benign decomposition products, which make them prospective and promising candidates for smokeless propellants, gas generators, and novel low-sensitivity high-energy explosives [1,2,3,4]. Energetic nitrogen-rich ionic salts are classified as high nitrogen energetic materials. These salts behave superior to atomically similar nonionic compounds in that they often possess negligible vapor pressures, lower melting points, higher densities, and better cohesive energy densities, which provide the best agreement between energy and sensitivity [5,6,7,8]. In principle, energetic nitrogen-rich ionic salts, a newly developing branch of energetic materials, have received a surge of attention in the past few years.

The present research for energetic ionic salts concentrates on synthesizing energetic anions and searching for desirable cations. The combination of different potential anions and cations gives a rich body of useful energetic ionic salts. The furazan, triazole, tetrazole, tetrazine, and pyrazine ring are effective fragments to construct energetic compounds owing to their inherent high nitrogen content and large positive heats of formation (HOFs) [9,10,11,12,13,14]. Among the NHEC community, furazanopyrazine derivatives stand out with excellent performance and good stability [15,16,17]. On the one hand, the absence of an acidic proton and electron-withdrawing character of furazan ring make it impossible to act as an anion or cation [18,19]. Pyrazine ring in furazanopyrazine derivatives is known as a dibasic acid [20], indicating that both hydrogen atoms on the pyrazine ring are acidic. Therefore, the combination of furazan with pyrazine ring deprotonates viable anions. On the other hand, there is a big π-conjugated molecular system in furazanopyrazine anions, resulting in thermally stable and insensitive ionic salts. 

4,8-dihydrodifurazano [3,4-*b*,*e*]pyrazine (DFP) is one kind of skeleton compound with two furazan rings fused by a pyrazine ring [21,22]. The presence of multiple C–N, N–O and C=N bonds contribute directly to its high enthalpy of formation. Evidently, DFP is a symmetric planar molecule with a high nitrogen content and a surprisingly high density of 2.01 g·cm^−3^ [15,23]. The parent difurazanopyrazine ring can be manipulated to obtain a desired set of properties by tailoring the structures of anions and cations. Introducing energetic group into molecular skeleton has long been an efficient strategy to improve the energetic performance of NHECs. The selection of different substituents and their positions on the ring have a profound influence on many physical properties, such as melting point, boiling point and viscosity [24,25,26,27,28,29]. As to the synthesis and properties of 4,8-dihydrodifurazano[3,4-*b*,*e*]pyrazine-based derivatives, some earlier work has touched on it. Liu et al. reported six difurazanopyrazine compounds containing different substituents and most of them are powerful and promising explosives with satisfactory oxygen balance and high density [23]. Li and his coworkers prepared a series of tetrazole-linked 4,8-dihydrodifurazano[3,4-*b*,*e*] pyrazine-based energetic salts with nitrogen-rich cations [30]. Therein, a unique ionic salt composed of diaminoguanidinium cation and 4,8-di(1*H*-tetrazol-5-yl)-difurazanopyrazine anion was proved to be potential high energy density material (HEDM) with outstanding thermal stability and desirable detonation performance comparable to well-known explosive 1,3,5,7-tetranitro-1,3,5,7-tetrazocane (HMX). However, systematic and comparative study on 4,8-dihydrodifurazano[3,4-*b*,*e*]pyrazine-based energetic salts is still lacking.

**Figure 1:**
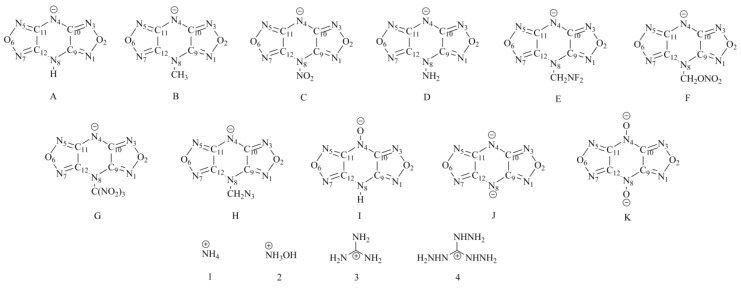
Frameworks of ammonium-based 4,8-dihydrodifurazano[3,4-*b*,*e*]pyrazine-based salts.

On the basis of the above considerations, eleven series of new energetic salts were designed from DFP precursor as shown in Figure 1. The combination of 4,8-dihydrodifurazano[3,4-*b*,*e*]pyrazine-based anions containing different substituents (–CH_3_, –NO_2_, –NH_2_, –CH_2_NF_2_, –CH_2_ONO_2_, –C(NO_2_)_3_, –CH_2_N_3_) with ammonium-based cations was made to establish broad potential ionic salts and evaluate their performances. Density functional theory (DFT) and volume-based thermodynamics calculations were further performed to screen 4,8-dihydrodifurazano[3,4-*b*,*e*]pyrazine-based energetic salts with high energy and acceptable stability. The densities, HOFs, detonation properties, impact sensitivity and thermal dynamics of formation were investigated in detail. This work aims to investigate the important role of different substituents, energetic cations, N–O oxidation bond and electric charge of anions in the formulation of efficient high-energy ionic salts.

## 2. Results and Discussions

### 2.1. Crystal Density

**Figure 2:**
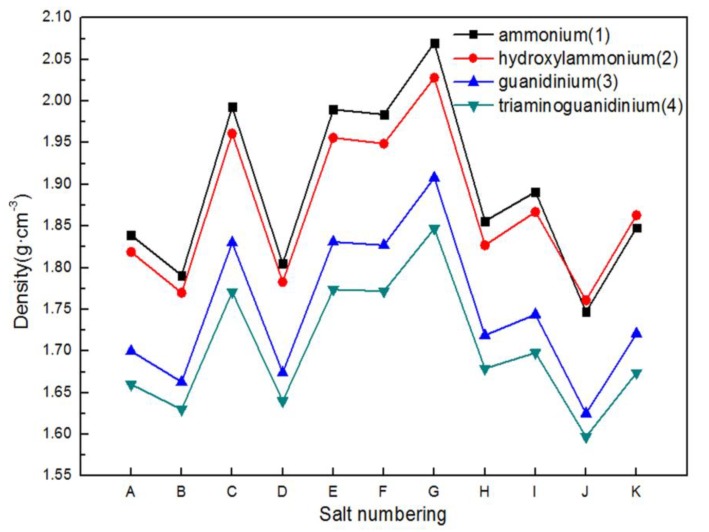
Comparison of the densities of ammonium-based 4,8-dihydrodifurazano[3,4-*b*,*e*]pyrazine-based salts.

According to Kamlet–Jacobs equation [31], the detonation velocity and pressure of an energetic compound are proportional to its density and a high density coming up with more energy packed in per unit volume is desirable. Our calculated densities and volumes of ammonium-based 4,8-dihydrodifurazano[3,4-*b*,*e*]pyrazine-based salts are listed in Table S1. Figure 2 presents a comparison of the densities of title salts.

From Table S1, it can be found that for 4,8-dihydrodifurazano[3,4-*b*,*e*]pyrazine-based anions A-H, the substitutions of group –NO_2_, –CH_2_NF_2_, –CH_2_ONO_2_, –C(NO_2_)_3_ make a significant increase of their densities as compared with the corresponding parent ones (A) for each cation series. This may be attributed to the following two aspects: One is that big π bond forms between difurazano[3,4-*b*,*e*]pyrazine ring and the substituent, resulting in a compact packing structure and thus a high density; the other is that the substituents enhance the mass of the salts markedly, but affect their volumes relatively little. Besides, the substitution of –CH_2_N_3_ slightly elevates the density values of the target salts. Among the substituted 4,8-dihydrodifurazano[3,4-*b*,*e*]pyrazine-based salts, the substitution with –C(NO_2_)_3_ exhibits the best performance. In particular, G1 and G2 feature such good density that even higher than 2.0 g/cm^3^ when compared with RDX (1.83 g/cm^3^) [32] and HMX (1.91 g/cm^3^) [33]. It can be concluded that –NO_2_, –CH_2_NF_2_, –CH_2_ONO_2_, –C(NO_2_)_3_ substituents are effective structural units to increase the densities of 4,8-dihydrodifurazano[3,4-*b*,*e*]pyrazine-based salts. However, when the H atom of the parent anion (A) is replaced by –CH_3_ or –NH_2_, the case is contrary and the densities of the corresponding salts are decreased. It could be explained that the H atoms of –CH_3_ and –NH_2_ are not coplanar with difurazano[3,4-*b*,*e*]pyrazine ring and hence cause a less efficient packing in crystal. One can find that the salts containing difurazano[3,4-*b*,*e*]pyrazine anion with one negative charge (A) possess higher densities than the ones with two negative charges (J). It may be caused by the two active positions on the structure of J anion that it can combine with more cations, resulting in a less dense packing and a lower density. In addition, the incorporation of N–O oxidation bond to the basic skeleton is favorable for increasing the densities of title salts when compared I and K series with A series. It is worth to note that the densities of K series with two N–O oxidation bonds are unexpectedly lower than those of I series with one N–O oxidation bond. It means that the effect of negative charge becomes predominant with regard to the densities of the title salts, which further confirms that increasing negative charge for 4,8-dihydrodifurazano[3,4-*b*,*e*] pyrazine anion is detrimental for improving the densities of title salts.

It can be seen from Figure 2 intuitively that all of the ammonium-based salts possess higher densities than those of guanidinium-based salts and the densities of triaminoguanidinium salts are the lowest among all the series. Therein, ammonium salts have the highest densities except J1 and K1. The densities of hydroxylammonium salts are a bit smaller than those of ammonium salts due to the volume-effect of hydroxy. It is interesting to find that the evolution pattern of the densities for different cation series is similar to the variation of the salt numbering. This shows that incorporating different cations could hardly alter the variation trend of density under the influence of different substituents.

### 2.2. Heats of Formation

Heat of formation (HOF) is another significant value for energetic materials because it is often considered as a reflection of energy content. Isodesmic reactions have been applied to calculate HOFs from *ab initio* calculations with reference to bond separation rules. Table S2 lists the calculated HOFs of reference molecules and ions in the isodesmic reactions along with available experimental values. From Table S2, we can find that all the relative errors are within 5%, indicating that the atomization approach to estimate HOFs of small molecules is reliable in this work.

Table S3 presents the HOFs of ammonium-based cations, 4,8-dihydrodifurazano [3,4-*b,e*]pyrazine-based anions and their corresponding salts, as well as lattice energies of these salts. It can be seen that the HOFs of all substituted 4,8-dihydrodifurazano[3,4-*b*,*e*]pyrazine salts (B-H) are higher than those of unsubstituted parent ones (A) with the same cation, implying that introducing the substituents into 4,8-dihydrodifurazano[3,4-*b*,*e*]pyrazine anion is favorable for advancing the HOFs of corresponding salts. Therein, the salts of G series with the substitution of –C(NO_2_)_3_ demonstrate the highest HOFs, followed by H series with –CH_2_N_3_ substituent. These observations confirm that –C(NO_2_)_3_ and –CH_2_N_3_ group contribute positively to the HOFs of 4,8-dihydrodifurazano[3,4-*b*,*e*]pyrazine-based energetic salts. Also, the salts containing two negative charges (J) have smaller HOFs than the parent ones (A) with one negative charge. Fortunately, involving N–O oxidation bond improves the HOFs of difurazano[3,4-*b*,*e*]pyrazine-based salts as compared I(K) with J series.

**Figure 3:**
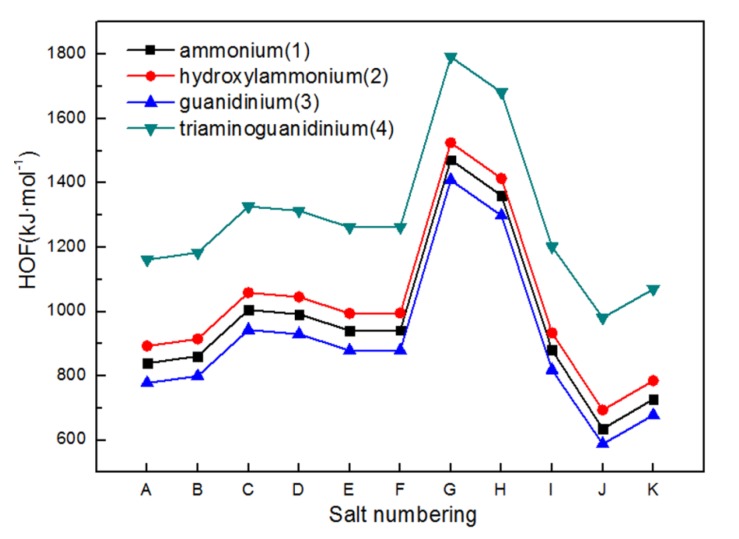
Comparison of the heats of formation (HOFs) of ammonium-based 4,8-dihydrodifurazano[3,4-*b*,*e*]pyrazine-based salts.

Figure 3 illustrates a comparison of the effects of different cations on the HOFs of title salts. As can be seen, guanidinium salts possess the smallest HOFs among all the salt series. Interestingly, when introducing –NH_2_ to guanidinium cation, the HOFs of title salts increase and triaminoguanidinium salts present the highest HOFs among all the anion series. Therefore, the HOF values of title salts decrease in the order of triaminoguanidinium salts > hydroxylammonium salts > ammonium salts > guanidinium salts. In addition, the evolution pattern of the HOFs for 4,8-dihydrodifurazano[3,4-*b*,*e*]pyrazine-based salts with different cations is consistent, suggesting that the nature of different substituents determines the HOF values of title salts, regardless of the variety of cations.

### 2.3. Energetic Properties

Detonation velocity and pressure are two important parameters reflecting the explosive performance for an energetic material. Table 1 gives a summary of the predicted densities (*ρ*), heats of formation (Δ*H*_f_), heats of detonation (*Q*), detonation velocities (*D*), detonation pressures (*P*), oxygen balance (OB), impact sensitivity (*H*_50_) and Gibbs free energies of reaction (Δ*G*_rxn_) for ammonium-based 4,8-dihydrodifurazano[3,4-*b*,*e*]pyrazine-based salts along with commonly used explosives RDX (1,3,5-trinitro-1,3,5-triazinane) and HMX. 

From Table 1, it can be found that when the substituent is –NO_2_, –NH_2_, –CH_2_NF_2_, –CH_2_ONO_2_, –C(NO_2_)_3_ or –CH_2_N_3_, its substituted difurazano[3,4-*b*,*e*]pyrazine salts exhibit higher heats of detonation than corresponding parent ones, whereas for the substituent –CH_3_, the case is quite the contrary. Among all the cation series, the substitution of –C(NO_2_)_3_ group owns the largest heat of detonation. In addition, the incorporation of N–O oxidation bond to difurazano[3,4-*b*,*e*]pyrazine-based salts promotes the heats of detonation to some extent. However, the increase of negative charge in difurazano[3,4-*b*,*e*]pyrazine anion is undesirable as the salts with two negative charges have evident lower heats of detonation than those with one negative charge.

As shown in Table 1, our calculated detonation properties of RDX and HMX agree well with experimental data, and thus the calculations for the energetic compounds are considered to be reliable. On the basis of the calculations by Kamlet–Jacobs equations, these energetic salts all have good detonation velocity from 7.21 to 10.50 km·s^−1^, detonation pressures from 21.91 to 54.02 GPa. The substituted 4,8-dihydrodifurazano[3,4-*b*,*e*]pyrazine-based salts possess higher *D* and *P* values than corresponding parent ones except for B series with the substitution of –CH_3_. One should note that the substitution of –C(NO_2_)_3_ presents the best detonation performance for each cation series owing to its high density and good heat of formation. It is observed that *D* and *P* values of C1, C2, E1, E2, F1, F2, and G1-G4 are very high and close to or above those of HMX. Therein, G1 presents the most excellent detonation velocity and pressure (*D* = 10.50 km·s^−1^, *P* = 54.02 GPa) among all the investigated energetic salts. This shows that –NO_2_, –CH_2_NF_2_, –CH_2_ONO_2_ or –C(NO_2_)_3_ group are effective substituents for improving the detonation performance of the title salts. Meanwhile, the introduction of N–O oxidation bond improves *D* and *P* remarkably compared I to A and K to J series. The salts containing difurazano[3,4-*b,e*]pyrazine anion with one negative charge have better detonation properties than the ones with two negative charges, suggesting that increasing negative charge for the anions is unreasonable for 4,8-dihydrodifurazano[3,4-*b*,*e*]pyrazine-based salts.

Oxygen balance (OB) is used to indicate the degree to which a compound can be oxidized and to classify energetic materials as either oxygen-deficient or oxygen-rich [34]. If an explosive has a negative oxygen balance, its combustion will be incomplete and a large amount of toxic gases such as carbon monoxide will be released. The higher the oxygen balance is, the larger the detonation velocity and pressure are and the better the performance of the explosive is. It can be seen from Table 1 that the substitution of –NO_2_, –CH_2_ONO_2_ and –C(NO_2_)_3_ group enhances OB values of the title salts significantly, suggesting that OB is greatly influenced by the number of nitro groups. However, when the substituent is –CH_3_, its substituted difurazano[3,4-*b*,*e*]pyrazine salts have lower OB than corresponding parent ones. Therein, hydroxylammonium salts possess the highest OB for each series, followed by ammonium salts. Unexpectedly, OB values of triaminoguanidinium salts are higher than those of guanidinium salts with the same anion. In addition, introducing N–O oxidation bond to difurazano[3,4-*b*,*e*]pyrazine anions is an effective method to enhance the OB values of title salts.

**Figure 4:**
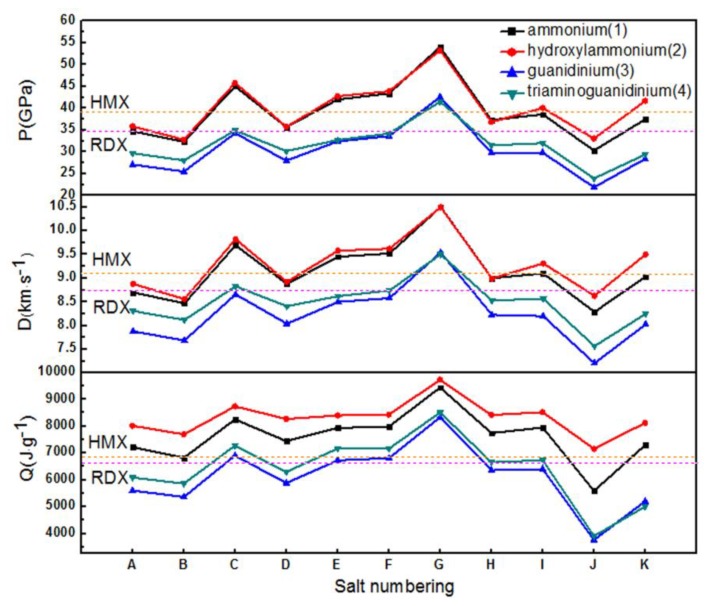
Comparison of the heats of detonation (*Q*), detonation velocities (*D*) and detonation pressures (*P*) of ammonium-based 4,8-dihydrodifurazano[3,4-*b*,*e*]pyrazine-based salts.

Figure 4 displays a comparison of the heats of detonation, detonation velocities and detonation pressures of ammonium-based 4,8-dihydrodifurazano[3,4-*b*,*e*]pyrazine-based salts. It is easily seen that ammonium-based salts have higher *Q*, *D* and *P* values than guanidinium-based salts with the same anion and most ammonium-based salts exhibit striking detonation performance approximate or surpass those of traditional powerful explosives RDX and HMX. The detonation velocity and pressure of title salts decrease in the order of hydroxylammonium salts > ammonium salts > triaminoguanidinium salts > guanidinium salts. Although the densities of hydroxylammonium and triaminoguanidinium salts are not outstanding, their high heats of formation compensate the disadvantage, proving again that the detonation performance of an energetic material is affected by both of density and heat of formation.

**Figure 5:**
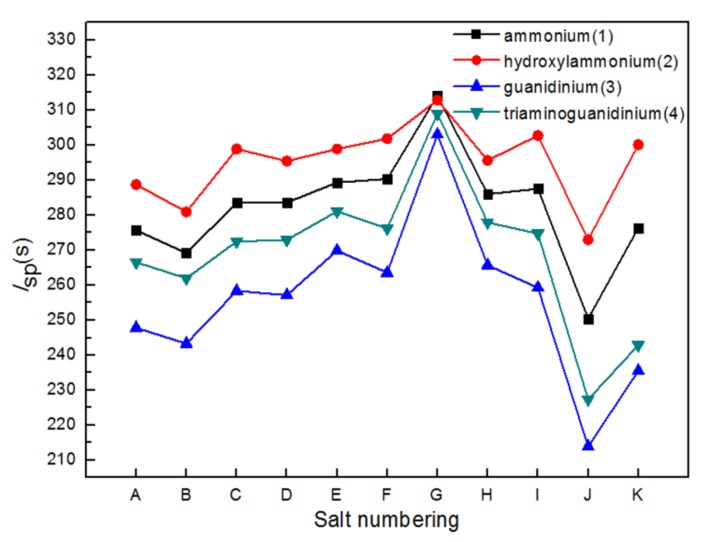
Comparison of the specific impulses (*I*_sp_) of ammonium-based 4,8-dihydrodifurazano[3,4-*b*,*e*]pyrazine-based salts.

Energetic salts are expected not only to be used as explosives, but also as components in advanced propellants. The specific impulse (*I*_sp_) is used to evaluate the performance of rocket propellant [35]. A comparison of specific impulses of ammonium-based 4,8-dihydrodifurazano [3,4-*b*,*e*]pyrazine-based salts was shown in Figure 5. When the substituent is –NO_2_, –NH_2_, –CH_2_NF_2_, –CH_2_ONO_2_, –C(NO_2_)_3_ or –CH_2_N_3_, their substituted difurazano[3,4-*b*,*e*]pyrazine salts have higher *I*_sp_ values than corresponding parent ones, whereas for the substituent –CH_3_, the case is quite the contrary. It is found that ammonium-based salts have greater *I*_sp_ values than guanidinium-based salts and hydroxylammonium salts exhibit the highest *I*_sp_ values for each anion series. The *I*_sp_ values of triaminoguanidinium salts are higher than those of guanidinium salts due to the increase of amino fragment. Furthermore, the incorporation of N–O oxidation bond is helpful for the increase of *I*_sp_ as compared I to A and K to J series. Overall, increasing negative charge is unfavorable considering its negative influence on *I*_sp_.

### 2.4. Impact Sensitivity

**Figure 6:**
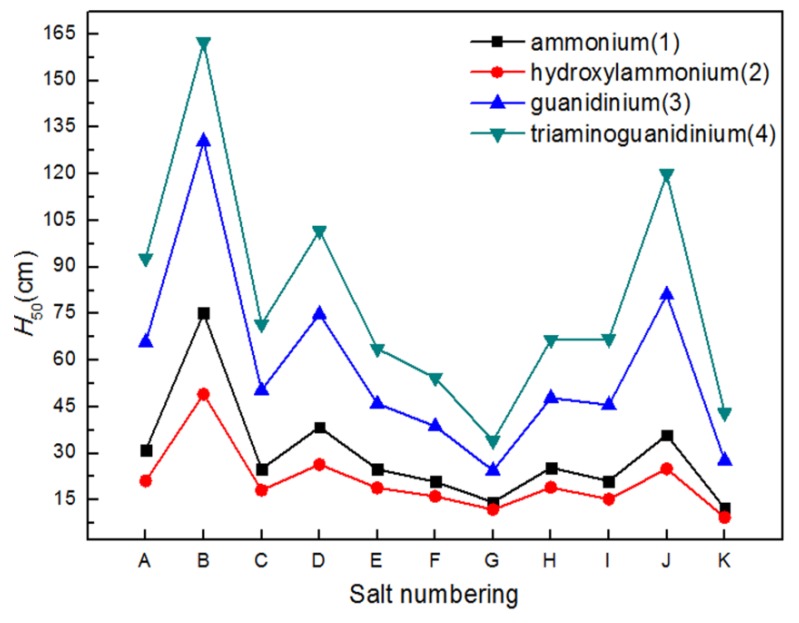
Comparison of the impact sensitivity (*H*_50_) of ammonium-based 4,8-dihydrodifurazano[3,4-*b*,*e*]pyrazine-based salts.

Impact sensitivity, one of the prime concerns in the field of energetic materials, is often characterized by impact drop height (*H*_50_, cm). *H*_50_ is the height from which there is a 50% probability of causing an explosion, where 1 cm = 0.245 J (Nm) with 2.5 kg dropping mass [36]. The impact sensitivity is inversely proportional to *H*_50_, namely, the higher *H*_50_ is, the less sensitive the explosive is [37]. Figure 6 illustrates a comparison of *H*_50_ of ammonium-based 4,8-dihydrodifurazano[3,4-*b,e*]pyrazine-based salts. As can be seen, guanidinium-based salts possess higher *H*_50_ values than ammonium-based salts, implying that incorporating more amino group into the cations is prone to the decrease of impact sensitivity. Therein, triaminoguanidinium salts exhibit the highest *H*_50_ values, and thus the lowest impact sensitivity. In addition, *H*_50_ values of B and E salt series are higher than those of corresponding parent ones, suggesting that –CH_3_ and –NH_2_ group are helpful for enhancing the stability of title salts. One should note that almost all the guanidinium-based salts possess *H*_50_ values approximate or higher than those of HMX and RDX. However, introducing the substituent –NO_2_, –CH_2_NF_2_, –CH_2_ONO_2_, –C(NO_2_)_3_, –CH_2_N_3_ and N–O oxidation bond into difurazano[3,4-*b,e*]pyrazine anion increases the impact sensitivity of title salts.

### 2.5. Gibbs Free Energies of Formation

Energetic ionic salts should not only have desirable explosive performance, but also be easy to synthesize. In this section, we predict the thermodynamics of formation of ammonium-based 4,8-dihydrodifurazano[3,4-*b*,*e*]pyrazine-based salts (Table S5). These salts were synthesized through two steps: One is the reactions of neutral substituted 4,8-dihydrodifurazano[3,4-*b*,*e*]pyrazine with potassium hydroxide to form nucleophilic nitrogen anions. The other is metathesis reactions of nitrogen anions with cation compounds to form the title salts. Therein, the key step is the first one.

**Figure 7:**
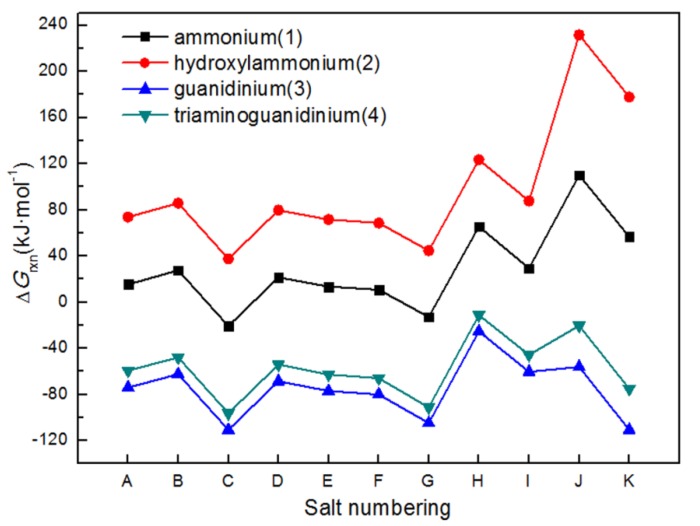
Comparison of Δ*G*_rxn_ of ammonium-based 4,8-dihydrodifurazano[3,4-*b*,*e*]pyrazine-based salts.

Figure 7 illustrates a comparison of the effects of cations and anions on thermodynamic data of Δ*G*_rxn_. It is obvious that all the guanidinium-based salts have negative Δ*G*_rxn_, indicating that the salts could be synthesized by the proposed reactions. In contrast, Δ*G*_rxn_ values of ammonium and hydroxylammonium salts, except for C1 and G1, are larger than zero. This means that the synthesis of these salts could not be easily realized. However, considering the computational uncertainty and the approximation of predicted phase transition, Dixon et al. believed that, if the free energy of reaction is positive and less than 41.8 kJ·mol^−1^, the synthesis of the salt may also be possible. Accordingly, it is inferred that a half of ammonium salts (A1, B1, D1, E1, F1, I1) and the hydroxylammonium salt-containing –NO_2_ substituent (C2) may also be synthesized, which is supported by experimental reports [23,30]. The possibility for the synthesis of other salts is lowered considerably by the designed reactions. Therefore, other feasible reactions need to be explored to synthesize these salts. Meanwhile, the substitution of –NO_2_ or –C(NO_2_)_3_ group decreases Δ*G*_rxn_ value for each cation series as compared with corresponding parent salts, implying that incorporating –NO_2_ group into anions is desirable for the synthesis of 4,8-dihydrodifurazano[3,4-*b*,*e*] pyrazine-based salts. Moreover, increasing negative charge in the difurazano[3,4-*b*,*e*]pyrazine anion improves the difficulty to synthesize corresponding salts. One encouragement is that for guanidinium-based salts, incorporating N–O oxidation bond into anions might be favorable on account of its positive influence on Δ*G*_rxn_ values of title salts.

### 2.6. Synthesis of Triaminoguanidinium 4,8-Dihydrodifurazano[3,4-b,e]pyrazine

**Scheme 1:**
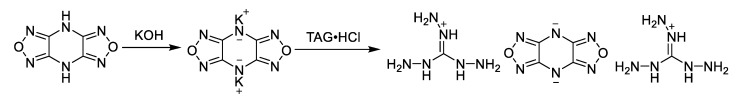
Synthetic route of J4.

With the properties given above, we performed an experimental investigation for J4 to test the accuracy of theoretical calculations. A synthetic route was designed and conducted for triaminoguanidinium 4,8-dihydrodifurazano[3,4-*b*,*e*]pyrazine (J4) as shown in Scheme 1.

The experimentally determined density of J4 is 1.602 g/cm^3^, which is approximate to its theoretical density value (1.597 g/cm^3^). Based on the experimental density and calculated heat of formation, detonation properties for J4 was determined by EXPLO5 (v6.02) program. It is found that J4 displays good detonation performance with detonation velocity of 7732 m/s and detonation pressure of 25.42 GPa, respectively, and thus outperforms TNT markedly. Further, the impact sensitivity test suggests that J4 is very insensitive with *H*_50_ value higher than 112 cm, which is consistent with calculated *H*_50_ value. The remarkable good stability of J4 could be rationalized that the delocalization of negative charges on the pyrazine ring strengthens bonds within the anions. These results confirm that the theoretical methods to predict the properties of 4,8-dihydrodifurazano[3,4-*b*,*e*]pyrazine-based energetic salts are reliable. The thermal stability of J4 was determined by differential scanning calorimetry (DSC) measurements. As can be seen from Figure S6, J4 begins to melt at 142.6 °C and exhibits good thermal stability with decomposition peak temperature at 216.7 °C. Given all the properties, J4 appears to be promising insensitive explosive.

## 3. Computational Methods

In this work, the calculations were carried out using Gaussian 09 (revision D.01) suit of programs. As far as we know, B3LYP/6-31++G* and MP2/6-311++G** are efficient and reliable basis sets for large molecules [41,42]. Therefore, the geometric optimization and frequency analyses were conducted at B3LYP/6-31++G* level and single-energy calculations at MP2/6-311++G** level. All the optimized structures were indicated for true local energy minima on the potential energy surfaces without imaginary frequencies.

### 3.1. Calculations of Density

Density is one of the most important physical properties of an energetic material. Several methods have been developed to accurately predict crystal density without a prior acknowledgement of the crystal structure [43,44,45]. With a complete neglect of intermolecular interactions within the crystal, conventional *M*/*V* approach to calculate densities leads to some large error greater than 0.05 g/cm^3^. An improved method by introducing electrostatic interaction correction to predict the crystal density of ionic crystals is shown in Equation (1) [46]:

(1) $$\rho =\partial \left(M/V\right)+\beta \left(V_{s}^{+}/A_{s}^{+}\right)+\gamma \left(V_{s}^{-}/A_{s}^{-}\right)+\delta$$

where *M* is the chemical formula mass of the compound, *V* is the volume of the isolated gas molecules defined as the space inside a counter of electron density of 0.001 e/Bohr^3^ using a Monte Carlo integration, *A*_S_^+^ is the portion of a cation’s surface which has a positive electrostatic potential, *V*_S_^+^ is the average value of a cation’s surface. *A*_S_^−^ and *V*_S_^−^ are the analogous quantities for an anion. The values of the coefficients *α*, *β*, *γ*, and *δ* are 1.026, 0.0514, 0.0419 and 0.0227, respectively [46]. Volume was calculated based on the optimized structures and the keyword “iop (6/46) = 2000” was used to perform 100 single-point calculations for each ion to minimize the random fluctuations and get an average volume. The surface electrostatic potential was obtained from Multiwfn program.

The volume of an ionic crystal with the formula M_p_X_q_ is straightforwardly the sum of volumes of the ions as shown in Equation (2) [47]:

(2) $$V=pV_{M^{+}}+qV_{X^{-}}$$

where *V*_M_^+^ and *V*_X_^−^ are the volumes of the cation M^+^ and anion X^−^, respectively. *p* and *q* are the number of cation M^+^ and anion X^−^ per formula unit.

### 3.2. Calculations of Heats of Formation

**Scheme 2:**
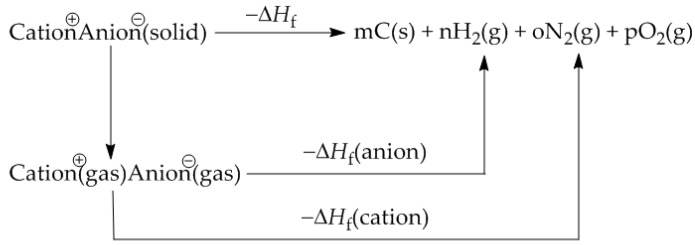
Born-Haber energy cycle for the formation of energetic salts.

On the basis of Born-Haber energy cycle (Scheme 2), the heat of formation of a salt could be evaluated according to Equation (3) [48]:

(3) $$\Delta H_{f}^{o}\left(\mathrm{salt},298K\right)=\Delta H_{f}^{o}\left(\mathrm{cation},298K\right)+\Delta H_{f}^{o}\left(\mathrm{anion},298K\right)-\Delta H_{L}$$

where Δ*H*_L_ is the lattice energy of the ionic salts, which could be predicted by the formula put up by Jenkins et al. as shown in Equation (4) [49]:

(4) $$\Delta H_{L}=U_{\mathrm{POT}}+\left[p\left(n_{M}/2\right)-2\right)]+[q\left(n_{X}/2\right)-2)]RT$$

where the values of *n*_M_ and *n*_X_ rely on the nature of the ions *M*_p_^+^ and *X*_q_^−^, respectively, and are equal to 3 for monatomic ions, 5 for linear polyatomic ions, and 6 for nonlinear polyatomic ions. *U*_POT_ (kJ/mol) is the lattice potential energy which could be estimated by Equation (5):

(5) $$U_{\mathrm{POT}}=\gamma {\left(\rho /M\right)}^{1/3}+\delta$$

where *ρ* (g/cm^3^) is the crystal density, *M* (g/mol) is the chemical formula mass of the ionic salt. For 1:1 (charge ratio) salts, the fitted coefficients *γ* and *δ* are 1981.2 kJ·cm/mol and 103.8 kJ/mol; for 1:2 salts, they are 8375.6 kJ·cm/mol and 178.8 kJ/mol; for 2:1 salts, they are 6764.3 kJ·cm/mol and 365.4 kJ/mol; for 2:2 salts, they are 6864.0 kJ·cm/mol and 732.0 kJ/mol, respectively [50,51].

**Scheme 3:**
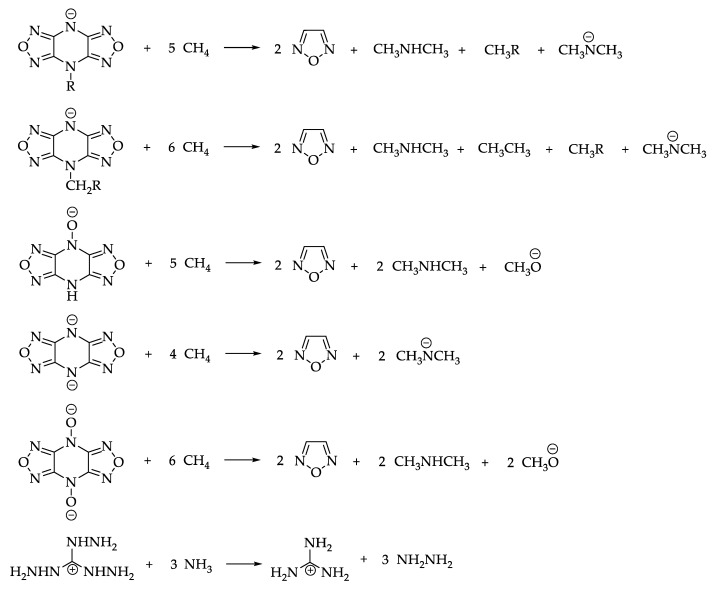
Isodesmic reactions for 4,8-dihydrodifurazano[3,4-*b*,*e*]pyrazine-based anions and ammonium-based cations.

The isodesmic reactions used for the prediction of gas-phase heats of formation (HOFs) of ammonium-based cations and 4,8-dihydrodifurazano[3,4-*b*,*e*]pyrazine-based anions at 298 K are shown in Scheme 3. HOFs of some small molecules whose experimental HOFs are unavailable were estimated by using atomization reactions [52,53]:

(6) $$C_{a}H_{b}O_{c}N_{d}\to aC\left(g\right)+bH\left(g\right)+cO\left(g\right)+dN\left(g\right)$$

### 3.3. Calculations of Energetic Properties

Detonation velocity (*D*) and pressure (*P*) can be estimated using empirical Kamlet–Jacobs equations as follows [31,54]:

(7) $$D=1.01{\left(NM^{1/2}Q^{1/2}\right)}^{1/2}\left(1+1.30\rho \right)$$

(8) $$P=1.558\rho^{2}NM^{1/2}Q^{1/2}$$

where *D* is the detonation velocity (km/s), *P* is the detonation pressure (GPa), *N* is the number moles of gaseous products per gram of explosive, *M* is the average molecular weight of gaseous detonation products, *ρ* is crystal density (g/cm^3^) and *Q* is the detonation energy (cal/g) which can be derived from HOFs of the products and reactants.

The oxygen balance (OB) value is also an important parameter for HEDMs. For an energetic compound C_a_H_b_O_c_N_d_, OB was calculated by the following equation [55]:

(9) OB=1600×(d−2a−b/2)/M×100%

The specific impulse (*I*_sp_) was used to evaluate the energy efficiency of propellant combustion. ISPBKW thermochemical code was applied to predict *I*_sp_ by considering ionic salts as monopropellants [38]. The equation for *I*_sp_ (s) is as follows [56]:

(10) $$I_{\mathrm{sp}}=9.33\sqrt{H_{c}-H_{e}}$$

where *H*_c_ is the sum of energies of combustion products at 68.0457 bars, *H*_e_ is the sum of energies of explosion products at 1.01325 bars.

### 3.4. Calculations of Impact Sensitivity

Michalchuk et al. proposed a vibrational up-pumping model based on vibrational energy transfer to predict impact sensitivity of an energetic material [57]. This method assumes that the impact excitation occurs via a conserved adiabatic heating process theoretically. Experimentally, impact sensitivity is characterized through a drop weight test. The drop height (*H*_50_) is defined as the height from which there is a 50% probability of initiating explosion. A simple method suggested by Keshavarz was used to predict *H*_50_ as follows [58,59]:

(11) $${\left(\log H_{50}\right)}_{\mathrm{core}}=-0.584+61.62a^{\prime }+21.53b^{\prime }+27.96d^{\prime }$$

(12) $$\left(\log H_{50}\right)={\left(\log H_{50}\right)}_{\mathrm{core}}+84.47F^{+}/MW-147.1F^{-}/MW$$

where (log*H*_50_)_core_ is the core function for estimation of impact sensitivity based on elemental composition; *a*’, *b*’, and *d*’ are the number of carbon, hydrogen, and nitrogen atoms divided by molecular mass of the energetic compound, respectively; *MW* is the molecular mass of the energetic compound. The data of *F*^+^ and *F^−^* are taken from Ref. [58].

### 3.5. Calculations of Gibbs Free Energies of Formation

The formation reaction of a salt is thermodynamically favorable when the free energy change, Δ*G*_rxn_, is less than or equation to zero. The generalized reaction for forming a salt is written as follows:

(13) anion base(gas)+cation base(gas)→{[cation][anion]}(salt)

**Scheme 4:**
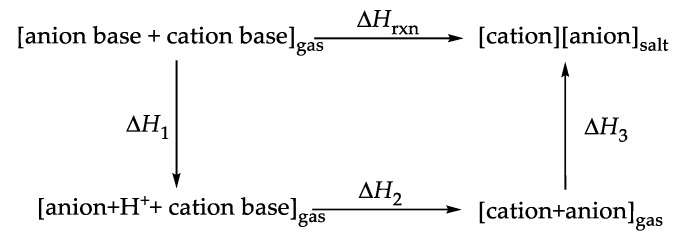
Born-Haber thermodynamic cycle for the formation of energetic salts.

The Born-Haber thermodynamic cycle for the formation of title salts is illustrated in Scheme 4. The total enthalpy of reaction (Δ*H*_rxn_) for producing the title salts is given by the enthalpy of each reaction, which is equal to the energy differences between a cation base and an anion base forming two ions in the gas phase, and plus the energy difference of two ions in the gas phase forming the salt in the solid phase. The total enthalpy of reaction was predicted by Equation (14) [12,27]:

(14) $$\Delta H_{\mathrm{rxn}}\left(\mathrm{salt}\right)=\Delta H_{1}+\Delta H_{2}+\Delta H_{3}$$

where Δ*H*_1_ is the enthalpy to dissociate the H–N bond in the anion base, Δ*H*_2_ is the affinity of the cation base for H^+^, Δ*H*_3_ is directly related to the lattice energy for salt formation. The enthalpies were obtained at the same level of theory as the single-point energy calculations.

The estimated entropy values (J·K^−1^·mol^−1^) of the title salts were calculated based on the relationship developed by Glasser and Jenkins for organic solids as shown in Equation (15) [60]:

(15) $$S_{298}^{o}=1.285\left(M/\rho \right)+57$$

where *ρ* and *M* are the same as employed in Equation (5) above. In the end, the total entropy of reaction (Δ*S*_rxn_) and the Gibbs free energies of formation (Δ*G*_rxn_) were calculated by the following two equations:

(16) $$\Delta S_{\mathrm{rxn}}\left(\mathrm{salt}\right)=S_{\mathrm{salt}}-S_{\mathrm{cationbase}}-S_{\mathrm{anionbase}}$$

(17) $$\Delta G_{\mathrm{rxn}}\left(\mathrm{salt}\right)=\Delta H_{\mathrm{rxn}}\left(\mathrm{salt}\right)-\Delta S_{\mathrm{rxn}}\left(\mathrm{salt}\right)$$

## 4. Materials and Methods

### 4.1. General Methods

DFP was synthesized according to the literature [61]. Triaminoguanidinium hydrochloride, absolute methanol and potassium hydroxide were bought from trade. ^1^H and ^13^C spectra were recorded on a Bruker 600 AVANCE spectrometer (Bruker, Switzerland). Infrared (IR) spectra (Nicolet, Madison; WI, USA) were collected on a NEXUS 870 spectrometer by Fourier transform techniques with KBr pellets. The spectrum was scanned in the range of 400–4000 cm^−1^and the resolution ratio is 4 cm^−1^. Elemental analysis (C, H, N) was performed with an Vario EL-III elemental analyzer. Densities were measured using a Micromeritics AccuPyc 1330 gas pycnometer (Micromeritics, Atlanta, GA, USA) at room temperature. Differential scanning calorimetery (DSC) was carried out with a NETZSCH5 instrument (NETZSCH, Selb, Bavaria, Germany). The sample (0.67 mg) was heated from room temperature to 400 °C at a heating rate of 10 °C/min in a nitrogen atmosphere. The impact sensitivity measurements were tested according to the Chinese GJB-772A-97 standard method 601.2 with a WL-1type impact sensitivity instrument [62]. The test conditions were as follows: Sample mass, 50 mg; drop weight, 2 kg.

### 4.2. Synthesis and Characterization of Energetic Materials

*Synthesis of 4,8-dipotassiumdifurazano[3,4-b,e]pyrazine (DFPK)* To a solution of DFP (0.66 g, 5.0 mmol) in absolute methanol (15 mL) at 10 °C, a solution of potassium hydroxide (0.62 g, 11.0 mmol) in water (1.5 mL) was added portionwise. The mixture was stirred for 0.5 h at room temperature. The precipitate was filtered off, washed with methanol and acetone, and dried to give 1.04 g yellow powder, yield 85.8%. ^13^C-NMR (D_2_O, 125 MHz), *δ*: 157.02; IR (KBr) cm^−1^: 1557 (C=N), 1532, 1381, 1341, 1011 (furazan ring), 887, 774; Anal. for C_4_K_2_N_6_O_2_ (mol. wt. 242.28): Calcd: C 19.83, N 34.69; Found: C 19.78, N 33.67. 

*Synthesis of triaminoguanidinium 4,8-dihydrodifurazano[3,4-b,e]pyrazine* Triaminoguanidinium hydrochloride solution (0.424 g, 3.0 mmol, 4 mL distilled water) was slowly added dropwise to a solution of DFPK (0.363 g, 1.5 mmol) in water (4 mL). The suspension was stirred for 2 h at room temperature. The precipitate was filtered off, washed with ice-water and dried to give 0.24 g yellow powder, yield 42.7%, and recrystallized from distilled water to give colorless needles. m.p. 142.6 °C, *T*_dec_ = 218.6 °C. ^1^H-NMR (DMSO-*d*_6_, 600 MHz), δ: 5.07 (s, 9H, NH–NH_2_); ^13^C-NMR (DMSO-*d*_6_, 125 MHz), *δ*: 152.84, 159.24; IR (KBr) cm^−1^: 3319, 3211 (NH_2_), 1557 (C=N), 1530, 1379, 1333, 1010 (furazan ring), 950, 886, 774; Anal. for C_6_H_18_N_18_O_2_ (mol. wt. 374.33): Calcd: C 19.25, N 67.35, H 4.85; Found: C 19.03, N 67.56, H 4.44.

## 5. Conclusions

In this work, a systematic and comparative study of densities, heats of formation, detonation performance, impact sensitivity, and Gibbs free energies has been performed for ammonium-based 4,8-dihydrodifurazano[3,4-*b*,*e*]pyrazine-based salts. It is found that –NO_2_, –CH_2_NF_2_, –CH_2_ONO_2_, –C(NO_2_)_3_ substituents are helpful for increasing the densities of 4,8-dihydrodifurazano[3,4-*b*,*e*] pyrazine-based salts and the substitution of –C(NO_2_)_3_ exhibits the highest density among all the cation series. Besides, the densities of ammonium-based salts are higher than those of guanidinium-based salts with the same anion. Incorporating the substituents into 4,8-dihydrodifurazano[3,4-*b*,*e*]pyrazine-based anions is helpful for improving HOFs of the title salts. However, one should be aware that increasing negative charge for difurazano[3,4-*b*,*e*]pyrazine anion is unfavorable for the improvement of densities and HOFs of title salts. 

All the substituents (except for –CH_3_) are helpful for enhancing the detonation performance and specific impulse of 4,8-dihydrodifurazano[3,4-*b*,*e*]pyrazine-based salts and the substitution of –C(NO_2_)_3_ presents the best performance. Therein, hydroxylammonium salts possess the highest detonation velocity and pressure due to their high density and good heat of formation. The calculations of *H*_50_ suggest that guanidinium-based salts possess lower impact sensitivity than ammonium-based salts and all their *H*_50_ values are higher than those of HMX or RDX.

The computational Gibbs free energies indicate that all the guanidinium-based salts could be synthesized by the proposed reactions. In the end, experimental work has been done for the energetic salt J4. The measurements of density and impact sensitivity for J4 support the validity of theoretical methods. It is anticipated that these findings will promote the future prospects for the rational design of nitrogen-rich energetic ionic salts.

## Figures and Tables

**Table 1:** Predicted densities (*ρ*), heats of formation (Δ*H*_f_), heats of detonation (*Q*), detonation velocities (*D*), detonation pressures (*P*), oxygen balance (OB), impact sensitivity (*H*_50_) and Gibbs free energies of formation (Δ*G*_rxn_) of ammonium-based 4,8-dihydrodifurazano[3,4-*b*,*e*]pyrazine-based salts.

Salts	*ρ* (g·cm^−3^)	Δ*H*_f_ (kJ·mol^−1^)	*Q* (J·g^−1^)	*D* (km·s^−1^)	*P* (GPa)	*I*_sp_ (s)	OB (%)	*H*_50_ (cm)	Δ*G*_rxn_ (kJ·mol^−1^)
A1	1.839	839.34	7224.4	8.70	34.69	275.69	−74.32	31.04	15.33
A2	1.819	892.94	8014.4	8.88	35.84	288.74	−60.30	21.13	73.82
A3	1.700	777.85	5602.5	7.88	27.10	247.82	−81.78	65.79	−73.96
A4	1.660	1161.66	6088.9	8.31	29.70	266.56	−77.04	92.83	−59.20
B1	1.791	861.46	6822.6	8.47	32.32	269.16	−93.40	75.11	27.67
B2	1.770	914.95	7696.0	8.56	32.77	280.93	−78.87	49.08	85.88
B3	1.663	799.61	5364.9	7.69	25.49	243.31	−97.07	130.44	−62.32
B4	1.630	1183.19	5864.2	8.12	28.04	261.99	−90.14	162.36	−47.87
C1	1.993	1005.48	8252.6	9.69	45.03	283.53	−42.11	24.98	−20.68
C2	1.961	1058.87	8736.4	9.82	45.80	298.89	−32.79	18.14	37.54
C3	1.830	943.42	6905.6	8.66	34.27	258.38	−53.33	50.38	−110.66
C4	1.771	1326.92	7278.4	8.83	34.91	272.51	−53.33	71.56	−96.22
D1	1.805	991.81	7446.4	8.88	35.70	283.61	−72.73	38.42	21.53
D2	1.783	1045.30	8269.2	8.92	35.74	295.46	−59.81	26.45	79.76
D3	1.674	929.97	5885.6	8.04	27.98	257.14	−80.00	74.85	−68.42
D4	1.640	1313.56	6300.9	8.41	30.20	272.95	−75.79	101.63	−53.95
E1	1.990	941.20	7934.8	9.45	42.02	289.33	−64.52	24.88	13.59
E2	1.956	994.52	8401.1	9.58	42.75	298.91	−54.55	18.87	71.61
E3	1.831	878.88	6726.2	8.50	32.41	269.88	−71.72	46.01	−76.90
E4	1.774	1262.19	7168.7	8.62	32.71	281.07	−69.25	63.72	−62.70
F1	1.984	942.08	7983.9	9.52	43.36	290.39	−49.61	20.85	10.76
F2	1.949	995.36	8430.0	9.62	43.87	301.83	−40.88	16.14	68.68
F3	1.827	879.64	6808.0	8.58	33.59	263.56	−58.67	38.72	−79.98
F4	1.772	1262.86	7160.3	8.74	34.21	276.18	−57.97	54.33	−65.89
G1	2.070	1471.80	9441.4	10.50	54.02	314.17	−19.28	14.29	−12.80
G2	2.028	1524.88	9725.2	10.49	53.25	312.85	−13.79	11.89	44.67
G3	1.908	1408.79	8332.8	9.54	42.61	303.05	−29.95	24.51	−104.67
G4	1.847	1791.64	8512.0	9.50	41.47	309.00	−32.46	34.02	−91.08
H1	1.856	1361.04	7745.3	9.00	37.30	286.03	−73.95	25.40	65.59
H2	1.827	1414.35	8419.0	8.99	36.86	295.66	−62.99	19.04	123.45
H3	1.719	1298.68	6360.7	8.23	29.79	265.72	−80.00	47.88	−25.26
H4	1.679	1681.93	6658.1	8.53	31.50	277.95	−76.31	66.60	−11.21
I1	1.891	880.26	7950.7	9.10	38.58	287.56	−60.30	21.13	29.35
I2	1.867	933.78	8522.8	9.31	40.04	302.73	−48.37	15.24	87.73
I3	1.744	818.54	6402.3	8.20	29.82	259.29	−69.71	45.57	−60.22
I4	1.698	1202.22	6735.2	8.57	32.01	274.76	−67.13	66.82	−45.60
J1	1.747	635.33	5590.5	8.29	30.31	250.45	−80.00	35.91	110.15
J2	1.761	693.94	7155.8	8.63	33.06	272.99	−55.17	25.06	231.71
J3	1.625	588.26	3771.2	7.21	21.91	213.91	−90.14	81.06	−56.11
J4	1.597	980.69	3911.9	7.57	23.91	227.36	−81.28	119.94	−20.34
K1	1.848	727.65	7301.0	9.03	37.45	276.35	−55.17	12.32	56.69
K2	1.863	785.27	8124.3	9.50	41.66	300.13	−36.36	9.42	177.74
K3	1.721	678.36	5204.0	8.03	28.39	235.55	−70.89	27.74	−110.66
K4	1.674	1070.13	5014.3	8.25	29.40	242.91	−67.00	42.99	−75.12
RDX	1.80	70.3	6656.7	8.87 (8.75 ^a^)	34.73 (34.70 ^a^)	290.52	−21.62	26.0 ^b^	—
HMX	1.91	116.12	6836.2	9.28 (9.10 ^a^)	39.19 (39.00 ^a^)	291.24	−21.62	29.0 ^b^	—

^a^ The experimental detonation velocities and detonation pressures (values in parentheses) for RDX and HMX are taken from Ref [38,39]. ^b^ The experimental data are taken from Ref [40].

## Supplementary Materials

The following are available online. Tables S1–S5: The predicted densities, HOFs, detonation properties and Gibbs free energies of 4,8-dihydrodifurazano[3,4-*b*,*e*] pyrazine-based salts. Figures S1 and S2: NMR spectra of 4,8-dipotassiumdifurazano[3,4-*b*,*e*]pyrazine, Figures S3 and S4: NMR spectra of triaminoguanidinium 4,8-dihydrodifurazano[3,4-*b*,*e*]pyrazine, Figure S5: IR spectra of J4, Figure S6: DSC curve of J4, Figure S7: Powder XRD patterns of DFP, TAG·HCl and J4.
