# Supplementary material for: Comparative Theoretical Studies on a Series of Novel Energetic Salts Composed of 4,8-Dihydrodifurazano[3,4-b,e]pyrazine-based Anions and Ammonium-based Cations
Source: Molecules. 2019 Sep 4;24(18):3213. doi: 10.3390/molecules24183213 (PMC6774342; doi:10.3390/molecules24183213)
Supplement: Supplementary file 1 [file molecules-24-03213-s001.pdf]

Supporting Information

Comparative Theoretical Studies on a Series of Novel Energetic Salts Composed of 4,8-Dihydrodifurazano[3,4-*b*,*e*]pyrazine-based Anions and Ammonium-based Cations

Binghui Duan[1], Ning Liu[1,2,*], Bozhou Wang[1,2], Xianming Lu[1,2], Hongchang Mo[1]

[1] Xi'an Modern Chemistry Research Institute, Xi'an 710065, People's Republic of China; duanbinghui@126.com

[2] State Key Laboratory of Fluorine & Nitrogen Chemicals, Xi'an 710065, People's Republic of China;

* Correspondence: (N.L.) flackliu@sina.com

Table of contents

Table S1. The calculated densities (*ρ*), volumes (*V*), the portions of ion's surface (*A*s) and the average values of electrostatic potential (*V*s) of ammonium-based 4,8-dihydrodifurazano[3,4-*b*,*e*]pyrazine-based salts.

Table S2. Calculated and experimental gas-phase HOFs (kJ·mol-1) for small molecules and ions at 298 K.

Table S3. The calculated heats of formation for ammonium-based cations, 4,8-dihydrodifurazano[3,4-*b*,*e*]pyrazine-based anions and their corresponding salts, and lattice energies of these salts.

Table S4. Predicted heats of detonation (*Q*), detonation velocities (*D*), detonation pressures (*P*), oxygen balance (*OB*) and impact sensitivities (H_{50}) of ammonium-based 4,8-dihydrodifurazano[3,4-*b*,*e*]pyrazine-based salts.

Table S5. Predicted entropies (S_{salt}), entropies of reaction (ΔS_{rxn}), enthalpies of reaction (ΔH_{rxn}) and free energies of reaction (ΔG_{rxn}) of ammonium-based

4,8-dihydrodifurazano[3,4-*b*,*e*]pyrazine-based salts.

Figure S1 ^{1}H NMR spectra of 4,8-dipotassiumdifurazano[3,4-*b*,*e*]pyrazine

Figure S2 ^{13}C NMR spectra of 4,8-dipotassiumdifurazano[3,4-*b*,*e*]pyrazine

Figure S3 ^{1}H NMR spectra of triaminoguanidinium

4,8-dihydrodifurazano[3,4-*b*,*e*] pyrazine

Figure S4 ^{13}C NMR spectra of triaminoguanidinium

4,8-dihydrodifurazano[3,4-*b*,*e*] pyrazine

Figure S5 IR spectra of J4

Figure S6 DSC curve of J4

Figure S7. Power XRD patterns of DFP, TAG•HCl and J4

Table S1. The calculated densities (ρ), volumes (V), the portions of ion's surface (As) and the average values of electrostatic potential (Vs) of ammonium-based 4,8-dihydrodifurazano[3,4-*b*,*e*]pyrazine-based salts.

Salts	V ($cm^3 \cdot mol^{-1}$)	V_s^+	A_s^+	V_s^-	A_s^-	ρ ($g \cdot cm^{-3}$)
A1	113.62	171.66	47.53	-87.95	167.24	1.839
A2	120.73	152.50	61.68	-87.95	167.24	1.819
A3	141.24	122.38	96.60	-87.95	167.24	1.700
A4	170.70	101.21	141.33	-87.95	167.24	1.660
B1	126.19	171.66	47.53	-84.44	185.19	1.791
B2	133.30	152.50	61.68	-84.44	185.19	1.770
B3	153.81	122.38	96.60	-84.44	185.19	1.663
B4	183.27	101.21	141.33	-84.44	185.19	1.630
C1	129.77	171.66	47.53	-82.09	190.04	1.993
C2	136.88	152.50	61.68	-82.09	190.04	1.961
C3	157.39	122.38	96.60	-82.09	190.04	1.830
C4	186.85	101.21	141.33	-82.09	190.04	1.771
D1	125.69	171.66	47.53	-85.29	180.55	1.805
D2	132.80	152.50	61.68	-85.29	180.55	1.783
D3	153.31	122.38	96.60	-85.29	180.55	1.674
D4	182.77	101.21	141.33	-85.29	180.55	1.640
E1	141.58	171.66	47.53	-78.79	210.35	1.990
E2	148.69	152.50	61.68	-78.79	210.35	1.956
E3	169.20	122.38	96.60	-78.79	210.35	1.831
E4	198.66	101.21	141.33	-78.79	210.35	1.774
F1	147.95	171.66	47.53	-74.58	226.71	1.984
F2	155.06	152.50	61.68	-74.58	226.71	1.949
F3	175.57	122.38	96.60	-74.58	226.71	1.827
F4	205.03	101.21	141.33	-74.58	226.71	1.772
G1	181.75	171.66	47.53	-72.80	250.98	2.070
G2	188.86	152.50	61.68	-72.80	250.98	2.028
G3	209.37	122.38	96.60	-72.80	250.98	1.908
G4	238.83	101.21	141.33	-72.80	250.98	1.847
H1	146.84	171.66	47.53	-77.66	213.71	1.856
H2	153.95	152.50	61.68	-77.66	213.71	1.827
H3	174.46	122.38	96.60	-77.66	213.71	1.719
H4	203.92	101.21	141.33	-77.66	213.71	1.679
I1	119.86	171.66	47.53	-85.69	176.70	1.891
I2	126.97	152.50	61.68	-85.69	176.70	1.867
I3	147.48	122.38	96.60	-85.69	176.70	1.744
I4	176.94	101.21	141.33	-85.69	176.70	1.698
J1	131.04	171.66	47.53	-178.38	172.07	1.747

J2	145.26	152.50	61.68	-178.38	172.07	1.761
J3	186.28	122.38	96.60	-178.38	172.07	1.625
J4	245.20	101.21	141.33	-178.38	172.07	1.597
K1	141.91	171.66	47.53	-170.18	190.18	1.848
K2	156.13	171.66	61.68	-170.18	190.18	1.863
K3	197.15	171.66	96.60	-170.18	190.18	1.721
K4	256.07	171.66	141.33	-170.18	190.18	1.674

Table S2. Calculated and experimental gas-phase HOFs (kJ·mol-1) for small molecules and ions at 298 K.

Molecules	ΔH_{cal}	ΔH_{exp}	Relative error/%	Molecules/ Ions	ΔH_{cal}	ΔH_{exp}	Relative error/%
NH_2NH_2	110.78	110.40[a]	0.34	Furazan	199.48	196.27[a]	1.64
CH_4	−73.27	−74.60[a]	-1.78	CH_3OH	−199.54	−201.50[a]	-0.97
CH_3CH_3	−82.44	−84.00[a]	-1.86	NH_2OH	−41.92	−45.0[a]	-6.84
CH_3NO_2	−83.87	−81.00[a]	3.54	CH_3O^-	−139.44[b]	−138.74[c]	0.50
CH_3NF_2	−119.29	−115.23[a]	3.52	NH_4^+	638.61[b]	626.40[c]	1.95
CH_3NH_2	−23.20	−22.50[a]	3.11	NH_3OH^+	685.63[b]	664.40[c]	3.19
CH_3N_3	299.74	296.50[a]	1.09	$CH_3NCH_3^-$	106.28[b]	103.74[c]	2.45
CH_3ONO_2	−126.50	−124.40[a]	1.69	guanidine	24.23[b]	25.30[c]	-4.23
NH_3	−45.30	−46.10[a]	-1.74	guanidinium	586.37[b]	575.90[c]	1.82
CH_3NHCH_3	−18.13	−18.90[a]	-4.07	triaminogua nidinium	904.45[b]	890.30[c]	1.59
$CH_3C(NO_2)_3$	117.49	113.80[a]	3.24				

[a] The values were taken from Ref. [1][b] The values were calculated by protonation reactions: $CH_3O^- + H^+ \rightarrow CH_3OH$, $NH_3 + H^+ \rightarrow NH_4^+$, $NH_2OH + H^+ \rightarrow NH_3OH^+$, guanidine + $H^+ \rightarrow$ guanidinium. [c] The values were taken from Ref. [2,3].

Table S3. The calculated heats of formation for ammonium-based cations, 4,8-dihydrodifurazano[3,4-*b*,*e*]pyrazine-based anions and their corresponding salts, and lattice energies of these salts.

Salts	ΔH_f anion(kJ • mol^{-1})	ΔH_f cation(kJ • mol^{-1})	lattice energy(kJ •mol^{-1})	ΔH_f(kJ • mol^{-1})
A1	620.44	643.86	424.96	839.34
A2	620.44	696.13	423.63	892.94
A3	620.44	578.49	421.08	777.85
A4	620.44	959.72	418.50	1161.66
B1	641.15	643.86	423.55	861.46
B2	641.15	696.13	422.33	914.95
B3	641.15	578.49	420.03	799.61
B4	641.15	959.72	417.68	1183.19
C1	784.63	643.86	423.01	1005.48
C2	784.63	696.13	421.89	1058.87

C3	784.63	578.49	419.70	943.42
C4	784.63	959.72	417.43	1326.92
D1	771.54	643.86	423.59	991.81
D2	771.54	696.13	422.37	1045.30
D3	771.54	578.49	420.06	929.97
D4	771.54	959.72	417.70	1313.56
E1	719.21	643.86	421.87	941.20
E2	719.21	696.13	420.82	994.52
E3	719.21	578.49	418.82	878.88
E4	719.21	959.72	416.74	1262.19
F1	719.53	643.86	421.31	942.08
F2	719.53	696.13	420.30	995.36
F3	719.53	578.49	418.38	879.64
F4	719.53	959.72	416.39	1262.86
G1	1246.61	643.86	418.67	1471.80
G2	1246.61	696.13	417.86	1524.88
G3	1246.61	578.49	416.31	1408.79
G4	1246.61	959.72	414.69	1791.64
H1	1138.67	643.86	421.49	1361.04
H2	1138.67	696.13	420.45	1414.35
H3	1138.67	578.49	418.48	1298.68
H4	1138.67	959.72	416.46	1681.93
I1	660.57	643.86	424.17	880.26
I2	660.57	696.13	422.92	933.78
I3	660.57	578.49	420.52	818.54
I4	660.57	959.72	418.07	1202.22
J1	786.59	643.86	795.12	635.33
J2	786.59	696.13	788.78	693.94
J3	786.59	578.49	776.82	588.26
J4	786.59	959.72	765.62	980.69
K1	875.13	643.86	791.34	727.65
K2	875.13	696.13	785.99	785.27
K3	875.13	578.49	775.26	678.36
K4	875.13	959.72	764.72	1070.13

Table S4. Predicted heats of detonation (*Q*), detonation velocities (*D*), detonation pressures (*P*), oxygen balance (OB) and impact sensitivities (H_{50}) of ammonium-based 4,8-dihydrodifurazano[3,4-*b*,*e*]pyrazine-based salts.

Salts	Q (J·g^{-1})	D (km·s^{-1})	P (GPa)	I_{sp} (s)	OB (%)	H_{50} (cm)
A1	7224.4	8.70	34.69	275.69	-74.32	31.04
A2	8014.4	8.88	35.84	288.74	-60.30	21.13
A3	5602.5	7.88	27.10	247.82	-81.78	65.79
A4	6088.9	8.31	29.70	266.56	-77.04	92.83

B1	6822.6	8.47	32.32	269.16	-93.40	75.11
B2	7696.0	8.56	32.77	280.93	-78.87	49.08
B3	5364.9	7.69	25.49	243.31	-97.07	130.44
B4	5864.2	8.12	28.04	261.99	-90.14	162.36
C1	8252.6	9.69	45.03	283.53	-42.11	24.98
C2	8736.4	9.82	45.80	298.89	-32.79	18.14
C3	6905.6	8.66	34.27	258.38	-53.33	50.38
C4	7278.4	8.83	34.91	272.51	-53.33	71.56
D1	7446.4	8.88	35.70	283.61	-72.73	38.42
D2	8269.2	8.92	35.74	295.46	-59.81	26.45
D3	5885.6	8.04	27.98	257.14	-80.00	74.85
D4	6300.9	8.41	30.20	272.95	-75.79	101.63
E1	7934.8	9.45	42.02	289.33	-64.52	24.88
E2	8401.1	9.58	42.75	298.91	-54.55	18.87
E3	6726.2	8.50	32.41	269.88	-71.72	46.01
E4	7168.7	8.62	32.71	281.07	-69.25	63.72
F1	7983.9	9.52	43.36	290.39	-49.61	20.85
F2	8430.0	9.62	43.87	301.83	-40.88	16.14
F3	6808.0	8.58	33.59	263.56	-58.67	38.72
F4	7160.3	8.74	34.21	276.18	-57.97	54.33
G1	9441.4	10.50	54.02	314.17	-19.28	14.29
G2	9725.2	10.49	53.25	312.85	-13.79	11.89
G3	8332.8	9.54	42.61	303.05	-29.95	24.51
G4	8512.0	9.50	41.47	309.00	-32.46	34.02
H1	7745.3	9.00	37.30	286.03	-73.95	25.40
H2	8419.0	8.99	36.86	295.66	-62.99	19.04
H3	6360.7	8.23	29.79	265.72	-80.00	47.88
H4	6658.1	8.53	31.50	277.95	-76.31	66.60
I1	7950.7	9.10	38.58	287.56	-60.30	21.13
I2	8522.8	9.31	40.04	302.73	-48.37	15.24
I3	6402.3	8.20	29.82	259.29	-69.71	45.57
I4	6735.2	8.57	32.01	274.76	-67.13	66.82
J1	5590.5	8.29	30.31	250.45	-80.00	35.91
J2	7155.8	8.63	33.06	272.99	-55.17	25.06
J3	3771.2	7.21	21.91	213.91	-90.14	81.06
J4	3911.9	7.57	23.91	227.36	-81.28	119.94
K1	7301.0	9.03	37.45	276.35	-55.17	12.32
K2	8124.3	9.50	41.66	300.13	-36.36	9.42
K3	5204.0	8.03	28.39	235.55	-70.89	27.74
K4	5014.3	8.25	29.40	242.91	-67.00	42.99
RDX	6656.7	8.87 (8.75[1])	34.73 (34.70[1])	290.52	-21.62	26.0[3]
HMX	6836.2	9.28 (9.10[2])	39.19 (39.00[2])	291.24	-21.62	29.0[3]

[1] The experimental detonation velocities and detonation pressures (values in parentheses) for RDX and HMX are taken from Ref [4,5].

[2] The experimental data are taken from Ref [6].

Table S5. Predicted entropies (S_{salt}), entropies of reaction (ΔS_{rxn}), enthalpies of reaction (ΔH_{rxn}) and free energies of reaction (ΔG_{rxn}) of ammonium-based 4,8-dihydrodifurazano[3,4-*b*,*e*]pyrazine-based salts.

Salts	S_{salt}	ΔS_{rxn}	ΔH_{rxn}	ΔG_{rxn}
	J·mol^{-1}·K^{-1}		kJ·mol^{-1}	
A1	184.87	-393.23	-101.85	15.33
A2	197.59	-412.21	-49.02	73.82
A3	227.05	-436.29	-203.97	-73.96
A4	265.97	-468.42	-198.79	-59.20
B1	198.35	-413.11	-95.44	27.67
B2	211.62	-431.52	-42.72	85.88
B3	241.68	-455.02	-197.92	-62.32
B4	280.85	-486.90	-192.97	-47.87
C1	204.01	-432.62	-149.60	-20.68
C2	216.92	-451.41	-96.98	37.54
C3	246.61	-475.26	-252.29	-110.66
C4	285.55	-507.38	-247.42	-96.22
D1	197.97	-411.02	-100.95	21.53
D2	211.20	-429.48	-48.23	79.76
D3	241.21	-453.02	-203.42	-68.42
D4	280.36	-484.92	-198.46	-53.95
E1	217.15	-452.55	-121.28	13.59
E2	230.46	-470.94	-68.73	71.61
E3	260.56	-494.38	-224.23	-76.90
E4	299.66	-526.33	-219.55	-62.70
F1	224.12	-466.58	-128.28	10.76
F2	237.65	-484.74	-75.77	68.68
F3	267.98	-507.96	-231.35	-79.98
F4	307.16	-539.83	-226.76	-65.89
G1	263.10	-507.85	-164.14	-12.80
G2	277.48	-525.15	-111.83	44.67
G3	308.83	-547.35	-267.78	-104.67
G4	348.45	-578.78	-263.56	-91.08
H1	221.77	-413.03	-57.50	65.59
H2	235.61	-430.88	-4.96	123.45
H3	266.27	-453.76	-160.49	-25.26
H4	305.66	-485.42	-155.87	-11.21
I1	192.19	-421.17	-96.16	29.35
I2	204.99	-440.06	-43.41	87.73

I3	234.56	-464.04	-198.51	-60.22
I4	273.49	-496.16	-193.46	-45.60
J1	205.48	-569.41	-59.54	110.15
J2	227.84	-610.43	49.81	231.71
J3	283.87	-661.50	-253.24	-56.11
J4	360.98	-726.48	-236.83	-20.34
K1	218.30	-607.85	-124.45	56.69
K2	239.08	-650.45	-16.10	177.74
K3	292.94	-703.69	-320.36	-110.66
K4	368.59	-770.14	-304.63	-75.12

^{1}H NMR (D_2O, 600 MHz)

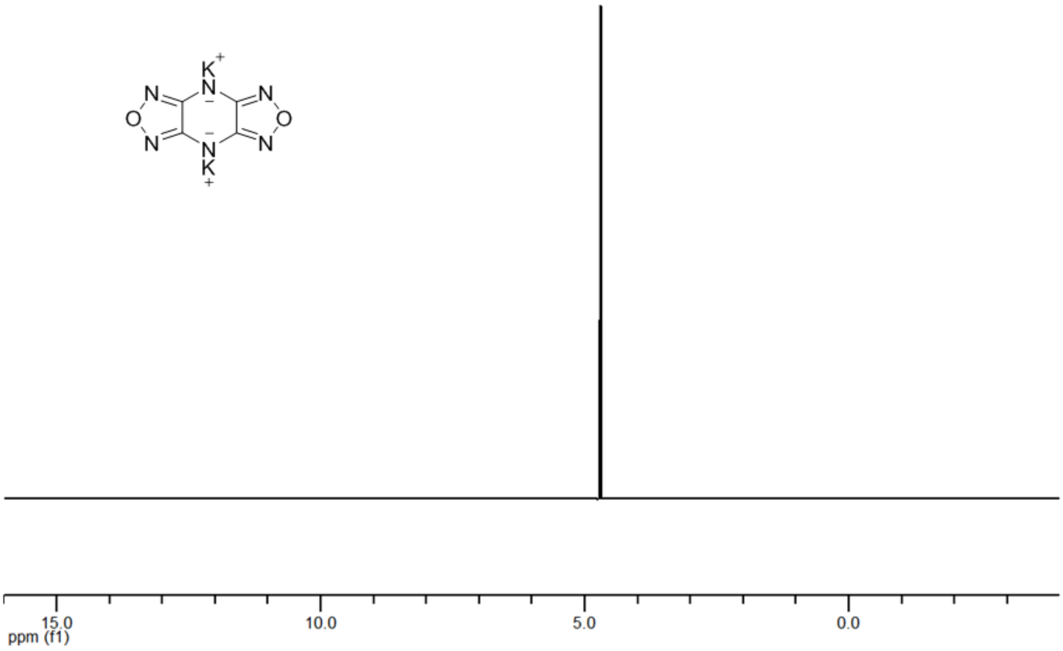

Figure S1. ^{1}H NMR spectra of 4,8-dipotassiumdifurazano[3,4-*b*,*e*]pyrazine.

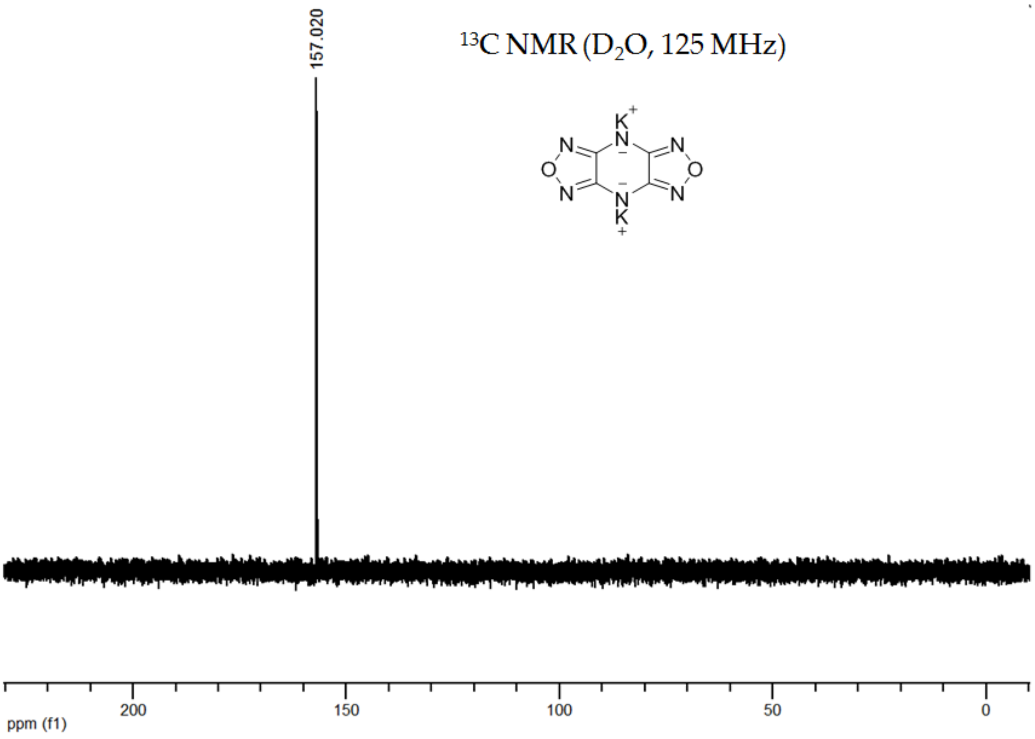

Figure S2. ^{13}C NMR spectra of 4,8-dipotassiumdifurazano[3,4-b,e]pyrazine.

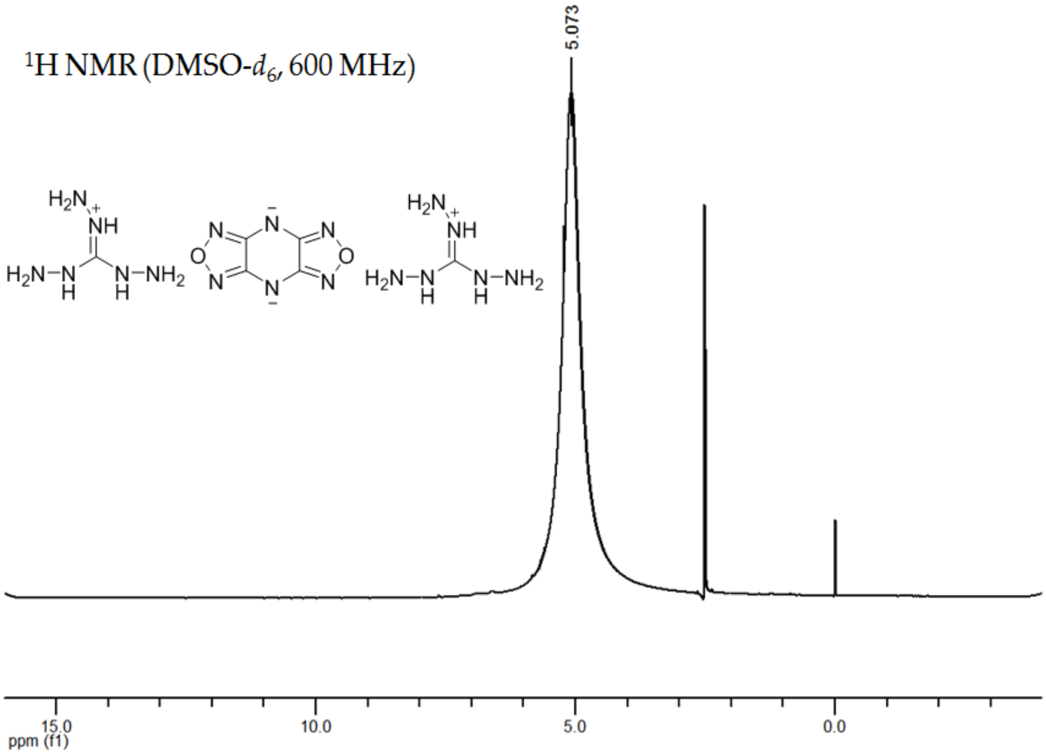

Figure S3. ^{1}H NMR spectra of triaminoguanidinium 4,8-dihydrodifurazano[3,4-*b*,*e*]pyrazine.

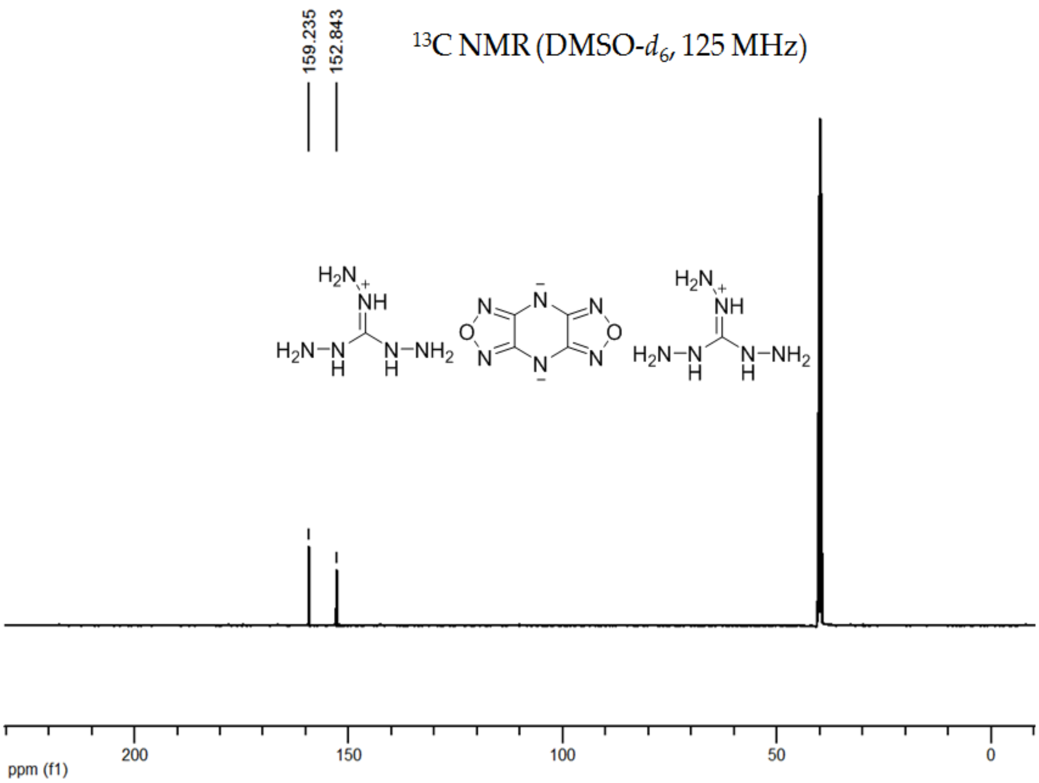

Figure S4. ^{13}**C** NMR spectra of triaminoguanidinium 4,8-dihydrodifurazano[3,4-*b*,*e*]pyrazine.

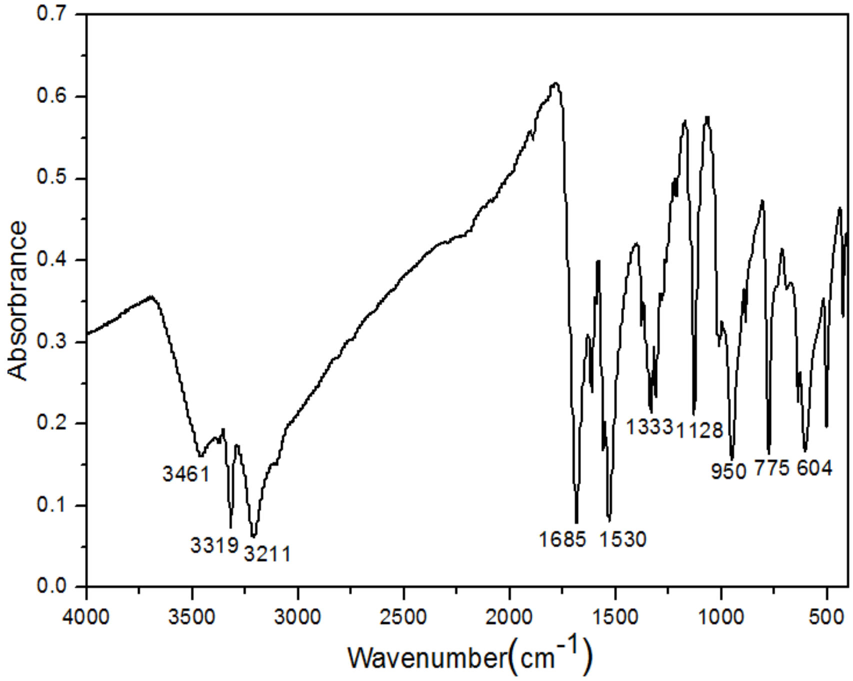

Figure S5. IR spectra of J4.

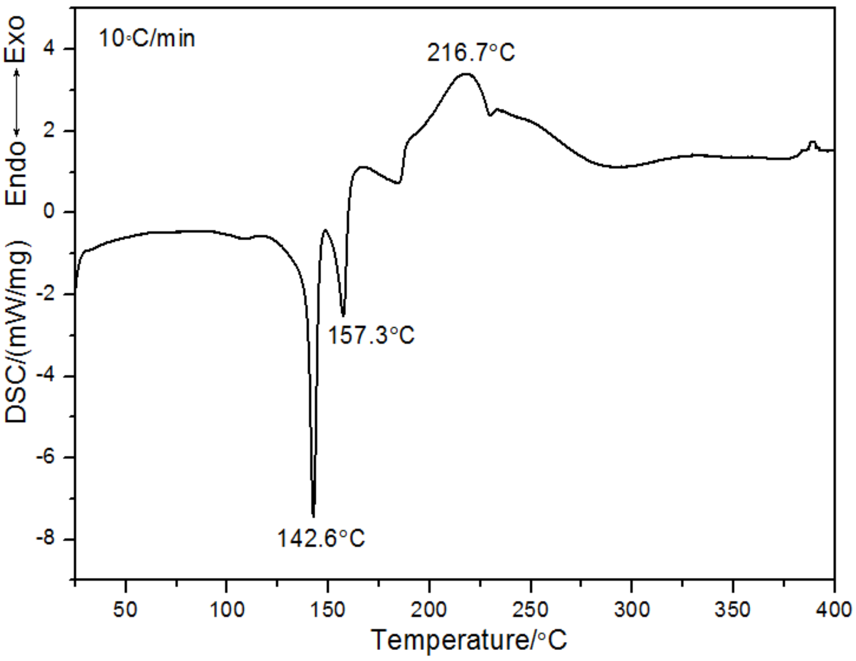

Figure S6. DSC curve of J4.

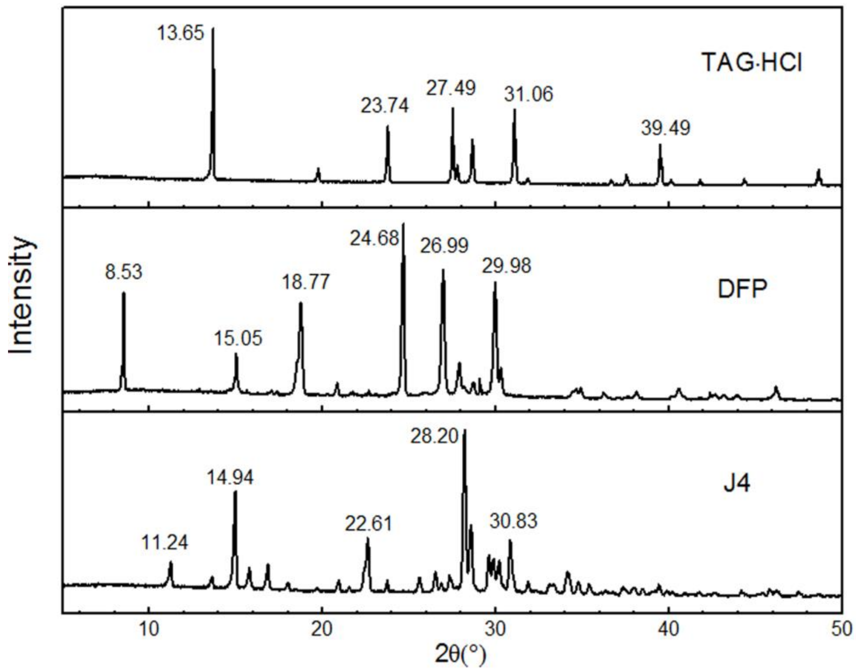

Figure S7. Powder XRD patterns of DFP, TAG ·HCl and J4.

References

1. Wei, T.; Zhang, J.J.; Zhu, W.H. A comparison of high-level theoretical methods to predict the heats of formation of azo compounds. *J. Mol. Struct.* **2010,** *956*, 55-60.
2. Jursic., B.S. Computing the heat of formation for cubane and tetrahedrane with density functional theory and complete basis set ab initio methods. *J. Mol. Struct.* **2000,** *499*, 137-140.
3. Lide, D.R., *CRC Handbook of Chemistry and Physics*. Boca Raton, **2004**.

4. Politzer, P.; Murray, J.S. Some perspectives on estimating detonation properties of C, H, N, O compounds. *Cent. Eur. J. Energy Mater.* **2011**, 8, 209-220.
5. Ou, Y.X.; Chen, J.Q. *High energy density compounds*; National Defence and Industrial Press: Beijing, China, **2005**.
6. Keshavarz, M.H.; Pouretedal, H.R.; Semnani, A. Novel correlation for predicting impact sensitivity of nitroheterocyclic energetic molecules. *J. Hazard. Mater.* **2007,** *141*, 803-807.
